# A Marriage Between Plastic Surgery and Nano-Medicine: Future Directions for Restoration in Mandibular Reconstruction and Skin Defects

**DOI:** 10.3389/fsurg.2020.00013

**Published:** 2020-03-27

**Authors:** Ava Brozovich, Elizabeth Andrews, Ennio Tasciotti, Jesse C. Selber

**Affiliations:** ^1^Texas A&M College of Medicine, Bryan, TX, United States; ^2^Department of Regenerative Medicine, Houston Methodist Research Institute, Houston, TX, United States; ^3^Orthopedics and Sports Medicine, Houston Methodist Hospital, Houston, TX, United States; ^4^Department of Plastic Surgery, MD Anderson, Houston, TX, United States

**Keywords:** mandiblar reconstruction, tissue regeneration, skin reconstruction, biomimctic materials, nanomedecine

## Introduction

Plastic and reconstructive surgery is one of the most diverse surgical sub-specialties. It encompasses a wide variety of techniques including grafts, flaps, free-tissue transfers, and replantation of various tissues ranging from nerve, vasculature, bone, muscle, and skin (1–3).

The goal in plastic surgery is to recreate both form and function of the resected or damaged tissue while maintaining or refining aesthetic appearance, and respecting blood supply. By adhering to these principles, the plastic surgeon is able to help optimize quality of life for the patient. Surgical techniques in general are largely focused on mechanical repair of tissue with less attention devoted to biological action. Despite incredible technical progress and innovations in surgical techniques, there are multiple defects and injuries that would benefit from stimulation of the underlying tissue biology to improve healing. Reconstructive surgery could greatly benefit from interplay with other fields of research such as bio- and nanomaterials. The field of nanomedicine is unique in that the size of the materials used allows researchers to have an effect on targeting and restoration at the molecular level (4, 5). The advantage of such materials is that the building blocks, due to their size and composition, have the ability to stimulate the repair of the body at a cellular level. More specifically, the objective is to develop a nano- and bio-materials whose structure and composition can mimic missing or damaged native biologic structures of the human body.

Though the traditional paradigm of tissue engineering includes the use of polymeric scaffolds, co-cultured cells, growth factors, extracellular matrix components, and other bioactive molecules, all integrated in a bioreactor, the field has recently seen the introduction of biomimetic materials as a novel way to elicit tissue response without the need of a bioreactor. Biomimetic materials capitalize on the body's intrinsic mechanisms of healing due to their ability to trigger the physiologic response of tissue specific resident cells tissue to undergo repair. The incorporation of nanomedicine principles in the design of tissue engineering grafts shifts the focus of regeneration to what is occurring at the cellular and molecular level as opposed to the larger spatial defect.

The marriage of nanomedicine and biomimetic tissue engineering presents a unique opportunity for the field of plastic and reconstructive surgery. A synergistic approach using both mechanical based reconstruction and restoration of damaged tissues with the help of biomimetic materials could be a major step forward in the future of regenerative and restorative medicine. In this paper, we will highlight specific areas in which these two fields can synergistically to solve clinical problems for the benefits of patients. The fusion of the mechanical craftsmanship in plastic surgery and the restorative properties of innate biological processes through nano- and bio-materials has the potential to create superior surgical outcomes compared to the outcomes that either field would be able to achieve alone.

## Mandibular Reconstruction

Mandibular reconstruction is often necessary for repairing defects due to oncologic resection or trauma. Methods of reconstruction are determined based on location and size of the mandibular defect, however, defects ≥6 cm are typically reconstructed with vascularized bone (6). The free fibula osteocutaneous flap is the workhorse donor site for mandibular reconstruction due to its length, compatibility for endosteal implants, and potential for skin islands if soft-tissue reconstruction is needed (7–10). Currently, the use of computer-aided design/computer-aided manufacturing (CAD/CAM) has been shown to result in improved function, morphology, and accuracy for complex segmental mandibular reconstruction compared to traditional free-hand techniques (11, 12).

Two of the most important goals in mandible reconstruction are mechanical stability with precise bone replacement, and osteointegration at the contact points between the native and reconstructed mandible. Although CAD/CAM has increased the sophistication of precise bone replacement, it has no effect on osteogenesis and osteointegration besides more precise bone to bone contact (13). A general approach to regenerative medicine as it relates to mandibular reconstruction includes structurally repairing the original defect and incorporation of the graft through enhanced vascularization with the end goal of restoring form, function, and innate biological activity.

Researchers have already begun exploring a nano-materials approach to bone regeneration in an attempt to repair large defects in the jaw, however, previous studies indicate that the bony matrix does not seem to properly utilize exogenous growth factors. For example, without nanotechnology BMP2 (bone morphogenetic protein-2) has been used to help stimulate bone growth, but led to devastating side effects including cancer, spinal cord compression (due to soft tissue swelling), ectopic bone formation, elevated bone resorption from overstimulation of osteoclast activation, and induction of adiopogenesis instead of the desired osteogenic process (14–21) Nanotechnology can offer a more sophisticated angle by creating a scaffold seeded with polymeric nanoparticles to facilitate optimal vascularization and innervation ensuring the regenerated bone is functional and integrated with the original structure of the face. For example, by utilizing a nanoarray of gold, it is possible to immobilize BMP-2, allowing a controlled release of BMP2 during the bone regeneration process, while also minimizing the side effects (22). Therefore, by seeding the scaffold with signaling factors, we could be able to provide controlled spatial and temporal delivery of these signaling molecules. This would allow plastic surgeons to have additional control over the regeneration to ensure adequate vascularization and structure. Not only could release of molecules be optimized, but the implant could be manufactured so that different spatial areas of the implanted scaffold achieve a specific biological function by releasing factors that are specific to the particular cellular response needed in that area. The scaffold can be manufactured from a variety of tissues including elastin, type I collagen/hydroxyapatite, or type I collagen. The materials that the scaffold is made of provides the mechanical and chemical stimuli to recruit the bodies own cells to the site of repair, as well as differentiate into bone. By optimizing vascularization and placing a focus on the biological mechanism in tissue repair, while also perfecting the shape of the scaffold to fit the natural contour of the patient, the regenerated bone will be optimally integrated within the native tissue, allowing function and form to be significantly superior to current mandibular reconstruction treatment options.

## Burns and Skin Regeneration

One of the primary techniques utilized by plastic surgeons to reconstruct burns include skin grafts—either full-thickness skin grafts (FTSG) or split-thickness skin grafts (STSG). Because skin grafts are harvested from the donor site without a blood supply, recipient site vasculature and ability to undergo angiogenesis is critical to skin graft survival. FTSG include the entire epidermis and dermis whereas STSG consist of the epidermis and varying degrees of dermis (23). FTSGs are limited to small defects due to the need to primarily close the donor site. Though autografts are considered the gold standard for skin reconstruction, it is not always feasible to use an autograft due to limited quantity of tissue availability for larger defects. However, STSG can cover larger defects because the donor site is left with dermis components to heal secondarily.{Braza, 2020 #57} In addition, the STSG can undergo more contraction and color changes compared to FTSG making it a favored technique (22).

The use of various skin substitutes and other biological components to alleviate limitations of autografts is a promising area of research. For example, Recell®, a non-cultured autologous skin cell spray has been used successfully as an adjunct to skin grafting in treating more severe burns or used alone for treatment of donor-site wounds. The suspension contains all cell-types (keratinocytes, papillary dermal fibroblasts, Langerhans cells and melanocytes) which can aid in faster healing, regimentation, and less scaring (24). However, the introduction of non-autologous materials into the body may result in an immune response and for a variety of other reasons is less reliable compared to an autograft.

Various low-level efforts at tissue engineering have included cultured expanded cell lines of autologous keratinocytes. These can then be “re-applied” in various ways to the wound. The disadvantage of these techniques is that they lack dermis and adnexal structures, are limited in thickness, and lack structural integrity (25). Early efforts aimed at using bio-printing to create a skin substitute are underway but suffer from the same drawbacks. These techniques are relatively primitive in comparison to the capabilities in modern tissue engineering and there is a sizable cost associated with culturing cells and utilizing bio-printing technology (26). For example, there is a huge limitation of whether enough cells can be readily generated to bio-print the necessary skin constructs. However, the upside to using materials at the nanoscale is the possibility to repair wounds quicker and more efficiently due to stimulation of the body's innate regenerative processes.

Synthetically derived polymers, defined here as materials that have been cultured *ex-vivo* or derived from a source other than the patients themselves, are being used as commercially available skin substitutes. These skin substitutes are composed of various materials including porcine and bovine collagen, shark chondroitin, cadaveric dermis, neonatal foreskin, cultured autologous epidermis (CAE) keratinocyte sheets or cell spray, and fibroblasts seeded onto a 3D hyaluronic acid derived scaffold or synthetic polymer membrane (27). However, though these products do have natural components such as hyaluronic acid, they have limitations including poor vascularization, failure to integrate, scarring, and immune rejection due to the addition of various skin substitutes (27). In addition, they require the patient to have limited movement during treatment, which is not always practical (28–31). In contrast, naturally derived polymers could have superior clinical function because they are more biocompatible, have lower immunogenicity, and could be resorbed over time as the newly deposited tissue gets remodeled (26, 32).

The development of a 2D monolayer for skin regeneration is not a novel research aim from the regenerative medicine field. However, researchers have recently started to capitalize on biomaterials in order to regenerate all three layers of the human epidermis. Developing a 3D model of skin is challenging as it involves the incorporation of different cells such as endothelial cells, Langerhan cells, and melanocytes. By using nanoparticles to stimulate the cells within the human body, it is possible to allow specific cells to produce the building blocks needed for skin regeneration. This regenerated skin could have superior bio-compatibility, lower immunogenicity, and lead to superior outcomes.

In addition, future biomimetic tissue engineering strategies need to focus on regenerating skin that has optimal color and texture, minimal scarring and inflammation, appropriate vascularization, and incorporation of nerve regeneration to minimize sensory parenthesis. The main factors that need to be achieved for a 3D *in vivo* skin scaffold are biodegradable material, evasion of immune response, and inclusion of growth factors or biomimetic substances to induce angiogenesis (32, 33).

Utilizing techniques found in nanotechnology would be beneficial to create a scaffold that provides mechanical cues to the construct such as directionality, porosity, and fiber size. For example, a common technique in nanotechnology is electrospinning. This technique creates long nano-fibers that can be embedded with antimicrobials or anti-inflammatories. In addition, keratinocyte-targeted lipid nanoparticles have been developed that can facilitate delivery of oligonucleotides to a wound or keratinocyte damaged site. Plastic surgeons currently using tissue-engineered grafts for wound repair are having problems with getting the keratinocytes to attach securely to the wound bed. Nanotechnology and biomimetics could help improve graft-survival by stimulating basement membrane formation and angiogenesis via small molecule stimulation (33).

When the entire thickness of skin is damaged, there is a plethora of inflammatory cells that may clear nanoparticles that are needed for tissue repair. However, by modifying the surface of the nanoparticles, nanoparticles may avoid clearance by macrophages. Such modifications include using anti-microRNAS to increased Dicer, thereby downregulating p21-Waf1/Cip1 expression (34). P21-Waf1/Cip1 expression is important in the regulation of activated T-cells, and therefore by decreasing its expression the inflammatory response would also be decreased (35).

Finally, in nanomedicine, previous research has led to the development of nano-scale films that were able to sense the mechanical stimuli of and the forces acting on the skin. These films could potentially be used to monitor and map the spatiotemporal mechanical properties necessary for skin regeneration. In addition, the use of a film could be used to sense the healing process throughout the regeneration process in order to tailor drug or surgical treatments on a personalized medicine level. For example, if the film sensed that a patient was taking more time to heal a skin wound, additional drug treatments could be infused to continue the regeneration process. Serendipitously, nanowires provide a high degree of sensitivity to provide the appropriate tunability necessary for skin regeneration. The use of these films in plastic surgery would allow the physician to follow in real time the process of skin restoration to fine-tune the healing process and achieve superior outcomes.

## Conclusion

Though the field of plastic surgery has made impressive advancements, there are still many improvements that could be made. By capitalizing on the techniques found in nanomedine, surgeons could be better equipped to restore function and achieve superior outcome. By harnessing the expertise of tissue engineers, biologists, material scientists, and plastic surgeons, the ability to design materials, implants and drug-eluting nanoparticles that can be quickly translated to the clinic becomes a realistic goal in the future. Studies are needed in the pre-clinical realm to translate from bench to bedside in order to further understand how to apply nanomedicine to the field of plastic surgery. Ultimately, collaboration of experts in these different fields will allow plastic surgeons to transform and advance the field, while vastly improving surgical outcomes.

## Author Contributions

AB prepared the initial article. EA assisted in preparation of the article. ET and JS assisted in editing the article.

### Conflict of Interest

The authors declare that the research was conducted in the absence of any commercial or financial relationships that could be construed as a potential conflict of interest.
